# The challenges of open data for future epidemic preparedness: The experience of the 2022 Ebolavirus outbreak in Uganda

**DOI:** 10.3389/fphar.2023.1101894

**Published:** 2023-02-10

**Authors:** Francesco Branda, Ahmed Mahal, Antonello Maruotti, Massimo Pierini, Sandra Mazzoli

**Affiliations:** ^1^ Department of Computer Science, Modeling, Electronics and Systems Engineering (DIMES), University of Calabria, Rende, Italy; ^2^ Department of Medical Biochemical Analysis, College of Health Technology, Cihan University—Erbil, Erbil, Kurdistan, Iraq; ^3^ Department GEPLI, Libera Università Ss Maria Assunta, Rome, Italy; ^4^ EpiData.it, Bergamo, Italy; ^5^ Statistics and Big Data, Universitas Mercatorum, Rome, Italy; ^6^ STDs Centre, Santa Maria Annunziata Hospital, Florence, Italy

**Keywords:** Uganda, viral infections, Ebola virus, infection control, outbreaks, surveillance, epidemiology

## Abstract

On 20 September 2022, the Ministry of Health in Uganda, together with the World Health Organization—Regional Office for Africa (WHO AFRO) confirmed an outbreak of EVD due to Sudan ebolavirus in Mubende District, after one fatal case was confirmed. Real-time information are needed to provide crucial information to understand transmissibility, risk of geographical spread, routes of transmission, risk factors of infection, and provide the basis for epidemiological modelling that can inform response and containment planning to reduce the burden of disease. We made an effort to build a centralized repository of the Ebola virus cases from verified sources, providing information on dates of symptom onset, locations (aggregated to the district level), and when available, the gender and status of hospitals, reporting bed capacity and isolation unit occupancy rate according to the severity status of the patient. The proposed data repository provides researchers and policymakers timely, complete, and easy-accessible data to monitor the most recent trends of the Ebola outbreak in Ugandan districts with informative graphical outputs. This favors a rapid global response to the disease, enabling governments to prioritize and adjust their decisions quickly and effectively in response to the rapidly evolving emergency, with a solid data basis.

## 1 Introduction

During the emergence of a novel pandemic, real-world data (RWD) are fundamental for informing public health policy decisions and improving clinical trials. In particular, in the early stages, there is a need to gain fundamental knowledge about the epidemiological characteristics of a new infection, from transmission potential to natural history (Branda et al., 2022a; Branda et al., 2022b). As outbreaks grow, there is a need to predict disease dynamics, estimate potential burden, and evaluate interventions (Branda et al., 2023). In the next steps, attention turns to estimating vaccine efficacy and monitoring outbreaks and evolutionary dynamics (Branda et al., 2020).

Although the African regions face recurrent epidemics and other health emergencies every year, the capacity to implement and analyze complex surveys tends to be limited as funding for data collection competes with other pressing needs. In particular, fragility, conflict and violence (FCV) affect data collection in many ways. For example, data collection during conflicts is affected by poor roads, inadequate telecommunications infrastructure, and sometimes populations hostile to central government representatives that provide few essential public services. In other cases, risks in FCV countries are often high due to disease. In Somalia, for example, it was not possible to conduct a traditional household consumption survey, with interviews lasting several hours, because of the level of insecurity and the danger interviewers faced if they spent more than an hour with a household. During the Ebola crisis, interviewers could not travel and collect information from respondents with face-to-face interviews because of the risk of infection.

The rapid outbreak sequencing of Ebola virus in 2022 demonstrated that the resurgence of Sudan virus disease (SVD) is a major public health concern in Uganda. On 20 September 2022, Ugandan health authorities declared an outbreak of Ebola disease, caused by Sudan virus, following the confirmation of a fatal case in a young male resident of Ngabano village of Madudu sub-county in Mubende district (World Health Organization, 2022). On 11 January 2023, after 42 days with no new cases, the outbreak was declared over. A total of 164 cases (142 confirmed, 22 probable) and 77 deaths (55 among confirmed cases and 22 among probable cases) were reported from September 20 to 10 January 2023. Uganda has reported in its history four SVD outbreaks in 2000, 2011 and two in 2012, before the last one in 2022. It is therefore likely that filoviruses are present in the reservoir of wild animals in the region. Therefore, the risk of re-emergence of any filovirus through exposure to an animal host or from a persistent virus cannot be ruled out. More details on Ebola virus are given in the Appendix section.

As we have seen with COVID-19, a critical component of a coordinated response is the rapid sharing of research results and data. Although we are fortunate that the Ebola virus has been well studied and that countermeasures exist to prevent and treat the disease, it is an evolving situation and there is still much to learn in order to anticipate the epidemic. According to a publication by the Johns Hopkins Center for Health Security, the African continent is the least prepared to respond to health emergencies, treat the sick and protect health workers (Johns Hopkins Center for Health Security, 2022) and has the lowest capacity to provide critical and intensive care in the world (World Economic Forum, 2022). The weakness of the health system and the high prevalence of malnutrition, malaria, HIV/AIDS and tuberculosis pose additional challenges. Therefore, strengthening surveillance capacity (Hoogeveen and Pape, 2020) can help detect future outbreaks, preventing their further spread. Our study describes a real-time database that we created to support epidemiological understanding of the origins and transmission dynamics of the Ebola epidemic in Uganda in 2022 and highlights the importance of having open data to quickly plan effective control measures should this epidemic grow further in the future.

## 2 Methods

To support global response efforts, we build an epidemiological surveillance for Ebola continuously and systematically collects, compares and analyzes information on all cases of EVD infection reported by the World Health Organization - Regional Office for Africa (WHO AFRO) (World Health Organization Uganda, 2022). Updates are not always available on a daily basis because there is a lag between the date of disease onset, the date of detection, and the date of reporting, resulting in a delay in reporting. Delays in reporting have the potential to distort the incidence curve of the epidemic, and in turn, estimates of transmission potential, forecasts of the outbreak trajectory, and the impact of control interventions (Kelly-Hope, 2008; Reijn et al., 2011). In the context of Ebola, factors influencing reporting delays include i) difficulties in tracing and monitoring contacts for rapid case isolation, ii) deliberate attacks on healthcare workers and suspension of healthcare outreach, iii) resistance of sick individuals to seek medical care as soon as the symptoms start and iv) population displacements (Shearer, 2018).

**FIGURE 1:**
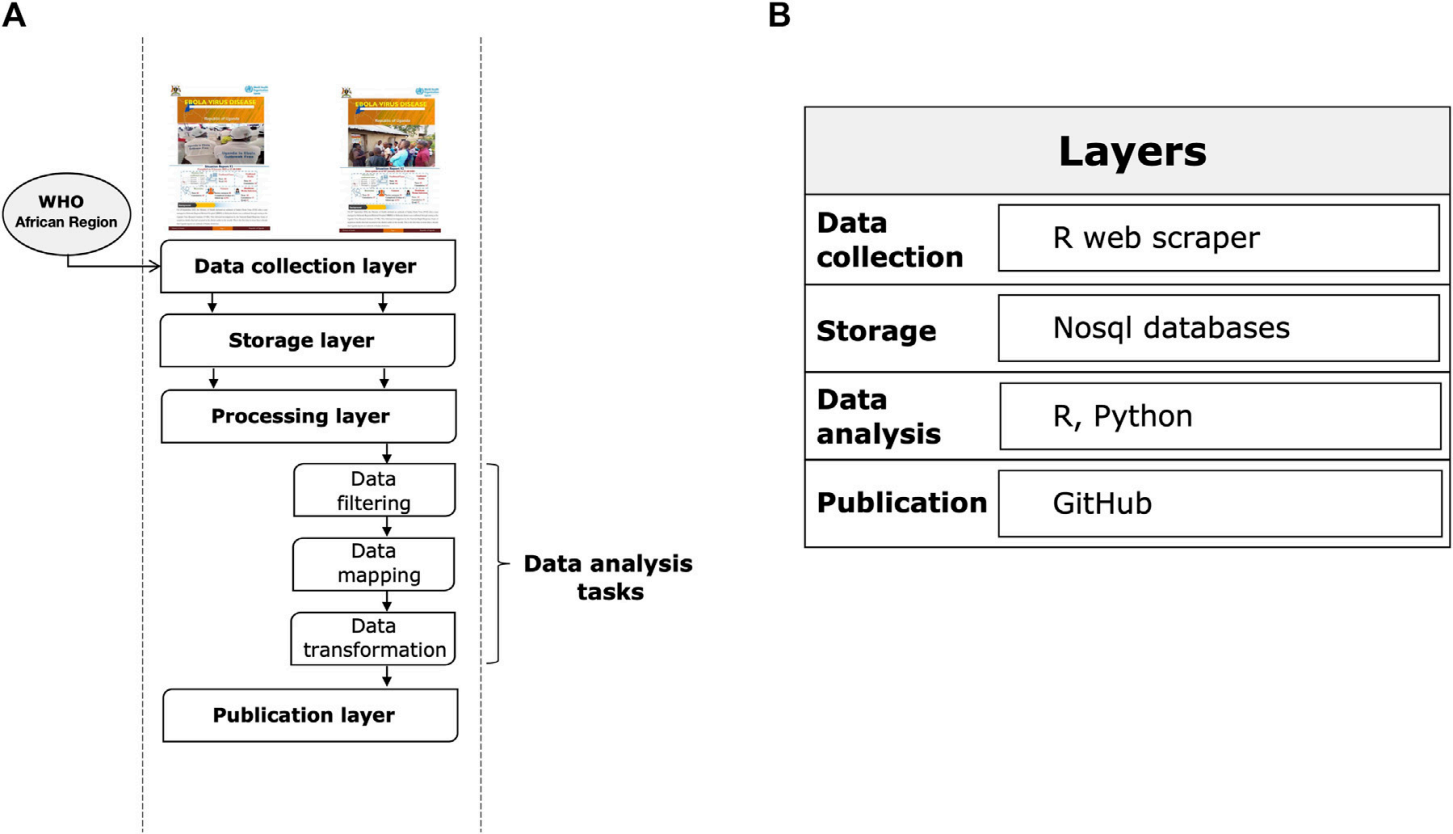
Layers of the Ebola information management system. **(A)** System execution flow. **(B)** Reference architecture.

The system consists of the steps described below (see Figure 1A): i) a *data collection layer* that collects shared data from verified sources, including reports from governments and public health organizations and statements from health officials reported in the media; ii) a *storage layer* that facilitates the storage and organization of data in an easily identifiable structure; iii) a *processing layer* that efficiently transforms, combines, and organizes data; iv) a *publication layer* that appropriately provides data and information to end users that they can use as a basis for epidemiological modeling to accelerate scientific discovery and response to the Ebola outbreak.

Figure 1B summarizes the main tools used for each step. The main types of data we collected using an automated web scraping in R: a) key dates, which include the date of laboratory confirmed cases, including infections among healthcare workers; b) demographic information about the sex of patients/cases; c) geographic information, at the highest resolution available down to the district level; d) any additional information such as the status of hospitals, i.e., the bed capacity and occupancy rate of isolation units according to the severity status of the patient. Note that point b) and d) are not always shared in public official reports. For the rapid evolution of the epidemic and a data pattern not defined *a priori* given the dynamic context, we have chosen to adopt a No-SQL approach for data storage. Data processing was conducted using several programming languages, including R and Python. Specifically, data engineering activities, such as resolving inconsistencies in text formats through conversion, string matching and manipulation, merging files, reorganizing folders, and maintaining archives and folder locations that contained the latest version of official reports, were performed using R packages. These activities were programmed to operate semi-automatically and required human supervision to monitor and perform quality checks. All processed data were analyzed daily by a dedicated team of epidemiologists, data scientists, and statistical experts through Python scripts. Data analysis focused primarily on trends, geo-spatial distribution, and epidemiological characterization of cases by disease severity and sex. Other types of analysis performed included risk profiling of Ugandan districts by outbreak intensity. Finally, Ebola data were published through a GitHub repository (https://github.com/fbranda/ebola).

## 3 Data description

Table 1 provides a short description of the database. In addition, the README file of the GitHub repository reports code snippets that can be used by a user to import such data into a variety of software programs.

**TABLE 1:** Database specifications.

Subject	Public health and health policy
Specific subject area	Infectious diseases and virology
Data accessibility	Public repository: GitHub (https://github.com/)
	Repository name: ebola
	Direct URL to data: https://github.com/fbranda/ebola
	License: CC-BY-4.0
Files and fields	1) Surveillance_data_Ebola_outbreak.csv
	• Date as of: Case reporting date
	• ConfCases: Daily number of new confirmed cases
	• CumCases: Cumulative number of confirmed cases
	• ConfDeaths: Daily number of new confirmed deaths
	• CumDeaths: Cumulative number of confirmed deaths
	• ConfRecoveries: Daily number of new confirmed recoveries
	• CumRecoveries: Cumulative number of confirmed recoveries
	• ConfHCWcases: Daily number of new confirmed cases of healthcare workers
	• CumHCWCases: Cumulative number of confirmed cases of healthcare workers
	• ConfHCWDeaths: Daily number of new confirmed deaths of healthcare workers
	• CumHCWDeaths: Cumulative number of confirmed deaths of healthcare workers
	2) Surveillance_data_Ebola_outbreak_by_district.csv
	• Date as of: Case reporting date
	• District: District name
	• ConfCases: Daily number of new confirmed cases
	• CumCases: Cumulative number of confirmed cases
	• ConfDeaths: Daily number of new confirmed deaths
	• CumDeaths: Cumulative number of confirmed deaths
	• ConfRecoveries: Daily number of new confirmed recoveries
	• CumRecoveries: Cumulative number of confirmed recoveries
	• ConfHCWcases: Daily number of new confirmed cases of healthcare workers
	• CumHCWCases: Cumulative number of confirmed cases of healthcare workers
	• ConfHCWDeaths: Daily number of new confirmed deaths of healthcare workers
	• CumHCWDeaths: Cumulative number of confirmed deaths of healthcare workers
	3) Surveillance_data_Ebola_outbreak_by_subcounty.csv
	• Date as of: Case reporting date
	• District: District name
	• SubCounty: Subcounty name
	• CumCases: Cumulative number of confirmed cases
	• CumDeaths: Cumulative number of confirmed deaths
	4) Surveillance_hospital_data_Ebola_outbreak.csv
	• Date as of: Case reporting date
	• Hospital: Hospital name
	•# of beds in the Isolation Unit: Cumulative number of beds occupied in the Isolation Unit (IU)
	•# of ETU beds: Cumulative number of beds occupied in the Ebola Treatment Units (ETU)
	•# of beds occupied in the Isolation Unit today: Daily number of beds occupied in the IU
	•# of beds occupied in the ETU today: Daily number of beds occupied in the ETU
	•# of suspect cases admitted to the Isolation Unit today: Daily number of suspect cases in the IU
	•# of Cases admitted to the ETU today: Daily number of cases in the ETU
	•# of walk in patients to the isolation Unit: Cumulative number of walk patients in the IU
	•# of Mild cases in the ETU today: Daily number of mild cases in the ETU
	•# of Critical cases in the ETU today: Daily number of critical cases in the ET
	•# of patients discharged from the ETU: Cumulative number of patients discharged from the ETU
	•# of patients discharged from the Isolation Unit: Number of patients discharged from the IU
	•# of suspect cases that died in the Isolation Unit: Number of suspect cases that died in the ETU
	•# of patients that died in the ETU: Number of patients that died in the ETU
	5) epicurve_by_notification_sex.csv
	• Date as of: Case reporting date
	• Sex: Sex of reported cases
	• ConfCases: Daily number of new confirmed cases
	• CumCases: Cumulative number of confirmed cases
	6) epicurve_by_onset_date.csv
	• Date as of: Case reporting date
	• Type of case: Type of case reported (confirmed/probable)
	• ConfCases: Daily number of new confirmed cases
	• CumCases: Cumulative number of confirmed cases

## 4 Usage notes

These data can be used to investigate the origins and transmission dynamics of the 2022 Uganda Ebola outbreak. This includes the estimation of key epidemiological parameters such as the incubation period and serial interval using mathematical models. Such models could be adapted to monitor the Ebola epidemic in other African regions, or for future outbreaks. In Supplementary material, we show a preliminary view of the collected epidemiological data and how they can be useful for direct visual assessment of the geographic distribution of risk areas as well as insights on the evolution of the outbreak over time. The data are openly available, and we will continue to curate the database as new information is made available.

While every effort has been made to standardize the data collected, some limitations must be recognized. The first is that although the data have been checked periodically wherever possible, conversion errors may occur when extracting data from the parent pdfs in machine-readable format. We have provided the sources consulted (i.e., the *Bulletins* folder in the GitHub repository) so that users can do further verification. There are then possible changes in reporting during the outbreak. For example, we found that demographic information or the status of hospitals reported initially were subsequently no longer made public. Although we have made every effort to report data as accurately as possible, given the dynamic nature of the outbreak, we caution that the database cannot be guaranteed to be error-free, and we apologize in advance if there are missing entries that were not detected using our standardized protocol. We invite database users to contact us directly if potential errors or omissions have been found. You can do so by emailing the corresponding authors or, preferably, by submitting a request *via* the Github repository.

## Data Availability

The original contributions presented in the study are publicly available. This data can be found here: https://github.com/fbranda/ebola.

## Appendix: Ebola virus disease

*Ebola* *virus* (EBOV) is a *Filovirus* involved in hemorrhagic, rare, high fatality rates and lack of effective treatment or vaccines, outbreaks in Sub-Saharan Africa. It recognizes probably in fruit bats Pteropodidae the reservoir animals, with spillovers in humans and primate apes (Taylor et al., 2010). Although there is evidence of wild mammals infected, the biology of host-filovirus interactions is not jet well understood (Emanuel et al., 2018), and it appears difficult to identify potential reservoir species with an expected long-term co-evolutionary history. The existence of filovirus-like elements, recorded as paleo viral, among mammalian genera, whose divergence dates have been estimated, suggests that filoviruses are at least tens of millions of years old (Emanuel et al., 2018), showing the possible co-existance of these viruses with humans and mammals from the beginning of their presence on Earth. Emerging hemorrhagic diseases has made the search for reservoir species a priority (Emanuel et al., 2018), seen the very high deaths rates: in some cases, the mortality in primates was so severe as to raise potentiality for extinction (Walsh et al., 2003).

*Filovirus* outbreaks are a known risk in Africa, with the first human case in 1976 (CDC. History, 2022) near the River Ebola in an area now known as the Democratic Republic of the Congo. Several outbreaks have been observed in recent years in other African countries. Here, we focus on the multiple outbreaks in Uganda where species of EBOV were observed over the last 20 years: i) *Sudan ebolavirus* (2000-2001, 2011, 2012, 2012/2013, 2022); ii) *Bundibugyo ebolavirus* (2007-2008); and iii) *Zaire ebolavirus* (2018-2020) which was imported from the Democratic Republic of the Congo (CDC. History, 2022).

This last 2022 EVD outbreak in Uganda is sustained by *Sudan ebolavirus;* no safe nor protective vaccine exists for this viral species. ERVEBO Vaccine, FDA approved, is protective only against *Zaire ebolavirus* species (CDC. History, 2022). Blood, secretions, organs, or other bodily fluids of dead or living infected people or animals contact are the dominant mode of transmission, but there is increasing evidence that different routes of transmission, including blood-borne, vertical, sexual, and aerosol transmission, can be impacting (MacIntyre and Chughtai, 2016). In recent years, a new paradigm of outbreaks has been suggested. It has been discovered that Ebola virus can be latent and persistent in infected persons and animals, with recovery of viral particles in human semen (EBOV RNA semen positive rate of 75.4% at 6 months from infection) (Thorson et al., 2021) and breast milk from women without previous infection (Sissoko et al., 2017). EBOV can reactivate in previous outbreaks survivors, also after long periods of time (Garry, 2022). This has been the starting event in recent Ebola virus Zaire species outbreaks in 2021 in Guinea [ (Keita et al., 2021)]. Suspected are small unrecognized chains of human-to-human transmission are believed to sustain the constant viral presence in the population in Guinea. This outbreak was not due to a new spillover from an animal reservoir but to the resurgence of latent Ebola virus particles, latent and persistent, in survivors: a reactivation. This new phenomenon epidemiologically implies detailed investigation of the index cases: in fact, latentization can be present in asymptomatic, pauci-symptomatic EBOV infections during previous outbreaks. Important is the survivor’s surveillance for monitoring eventual reactivations and relapses and viral strains genotyping and phylogenetic reconstruction. In the case of New Guinea Outbreak the index case was a nurse. The greatest risk of acquiring the infection is in healthcare workers due to direct contact with patients and/or local communities in affected areas. In addition, staff members of humanitarian, religious and other organizations, who have a large presence in the country, may be exposed to the virus, but the likelihood of infection for this group is considered low if infection prevention and control measures are followed.
