# Supplementary material for: The challenges of open data for future epidemic preparedness: The experience of the 2022 Ebolavirus outbreak in Uganda
Source: Front Pharmacol. 2023 Feb 10;14:1101894. doi: 10.3389/fphar.2023.1101894 (PMC9950500; doi:10.3389/fphar.2023.1101894)
Supplement: Supplementary file 1 [file DataSheet1.pdf]

*Supplementary Material*

# The challenges of open data for future epidemic preparedness: The experience of the 2022 Ebolavirus outbreak in Uganda

**Francesco Branda[1,*], Ahmed Mahal[2], Antonello Maruotti[3], Massimo Pierini[4,5], Sandra Mazzoli[4,6]**

[1]Department of Computer Science, Modeling, Electronics and Systems Engineering (DIMES), University of Calabria, Rende, Italy

[2]Department of Medical Biochemical Analysis, College of Health Technology, Cihan University—Erbil, Erbil, Kurdistan Region, Iraq

[3]Department GEPLI, Libera Università Ss Maria Assunta, Rome, Italy

[4]EpiData.it, Bergamo, Italy

[5]Statistics and Big Data, Universitas Mercatorum, Rome, Italy

[6]STDs Centre, Santa Maria Annunziata Hospital, Florence, Italy

*** Correspondence:**
Corresponding Author
francesco.branda@unical.it

## 1 Epidemiological summary

On January 11, 2023, Uganda's Ministry of Health (MoH) declared the end of the Ebola outbreak caused by Sudan ebolavirus (SVD) that affected nine districts. In accordance with WHO recommendations, the declaration was made 42 days (twice the maximum incubation period for *Sudan ebolavirus* infections) after the sample from the last hospitalized case, collected on November 29, 2022, tested negative before discharge, and the last confirmed death was granted a safe and dignified burial on November 29, 2022. This outbreak is not unexpected, as SVD is enzootic and present in animal reservoirs in the region. Uganda reported four outbreaks of SVD in 2000, 2011, and two in 2012 before this latest one in 2022, as well as two Ebola outbreaks caused by *Bundibugyo ebolavirus* in 2007 and *Zaire ebolavirus* in 2018 [1] (see Supplementary Figure 1).

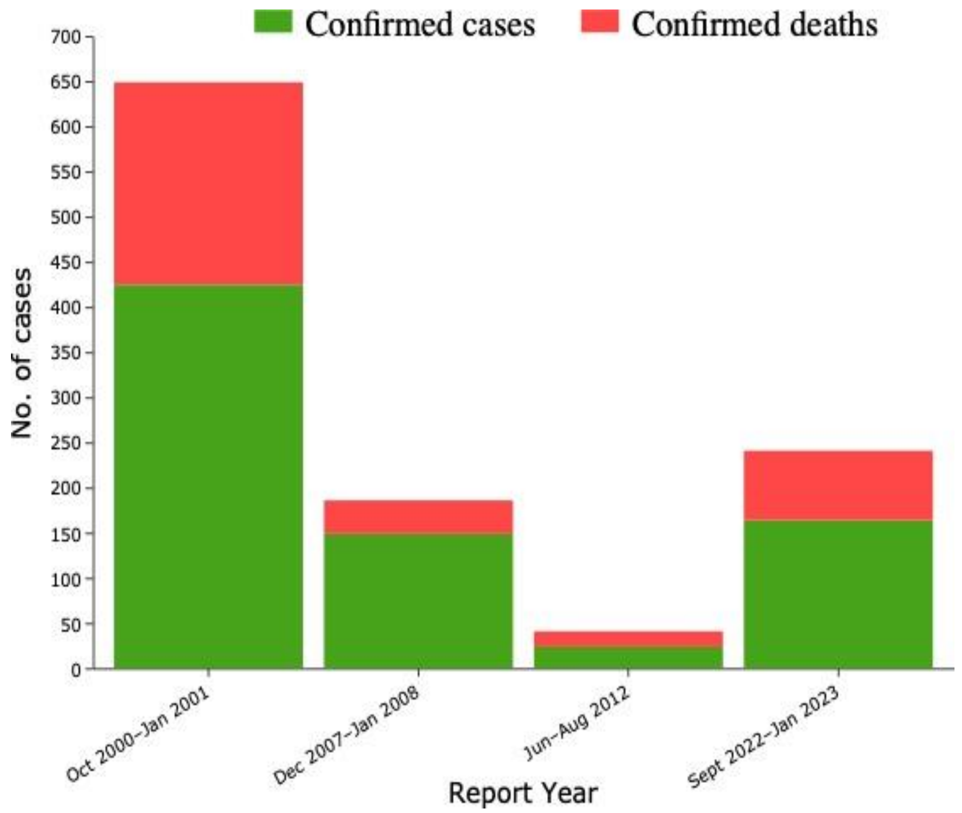


**Supplementary Figure 1. Major Ebola outbreaks in Uganda.**

## 2 Geographical distribution of Ebola virus cases in Uganda 2022

The first case was reported in the district of Mubende, but due to the lack of data it is difficult to locate the origin of this outbreak, whether due to an animal-to-human spillover or infection with infected biological materials of animal/human origin, or to other causes, including a possible human-to-human contagion or reactivation of a previous infection, as in the case of *Zaire ebolavirus*. Supplementary Figure 2 shows a preliminary view of the collected epidemiological data, providing some hints on

trends and data distribution. In particular, Supplementary Figure 2(A) shows the time plot of infection data, where the number of cases (daily confirmed cases and deaths) is plotted against the time of reporting. Supplementary Figure 2(B) shows the distribution of EVD cases by district. The plot immediately reveals some interesting features. The first important finding to emerge from the temporal analysis of the cases is that the epidemic appears to have started in Mubende in September and moved into neighboring districts with Kassanda the most affected. Second, a higher proportion of cases occurred among males.

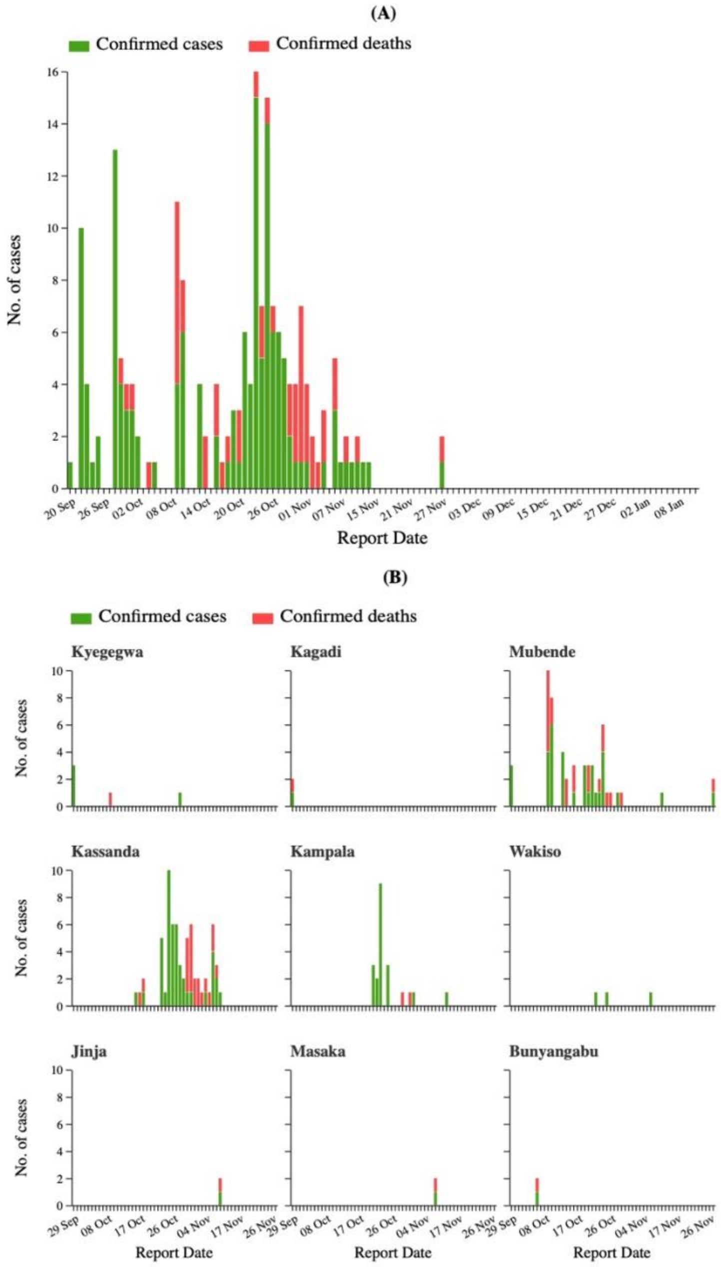


**Supplementary Figure 2. Ebola cases in Uganda 2022. (A) Daily number of new confirmed cases and deaths. (B) Time analysis of affected persons by district.**

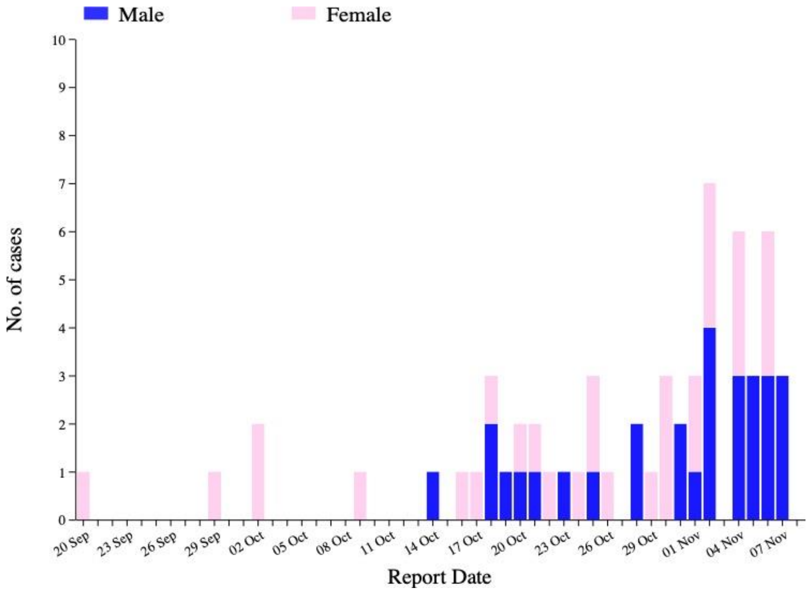


**Supplementary Figure 3. Daily number of new confirmed cases by sex.**

The most affected districts are shown in Supplementary Figure 4. Mubende was the epicentre of the outbreak, accounting for 45% of confirmed cases (64 confirmed and 19 probable), followed by Kassanda with 35% of confirmed cases (49 confirmed and 2 probable), Kampala with 13% of confirmed cases (17 confirmed and 1 probable), Wakiso with 3% of confirmed cases (4 confirmed cases), Kyegegwa with 2% of confirmed cases (3 confirmed cases), Jinja with 1% of confirmed cases (2 confirmed cases), and Bunyangabo, Kagadi and Masaka, which reported one confirmed case each (Supplementary Table 1).

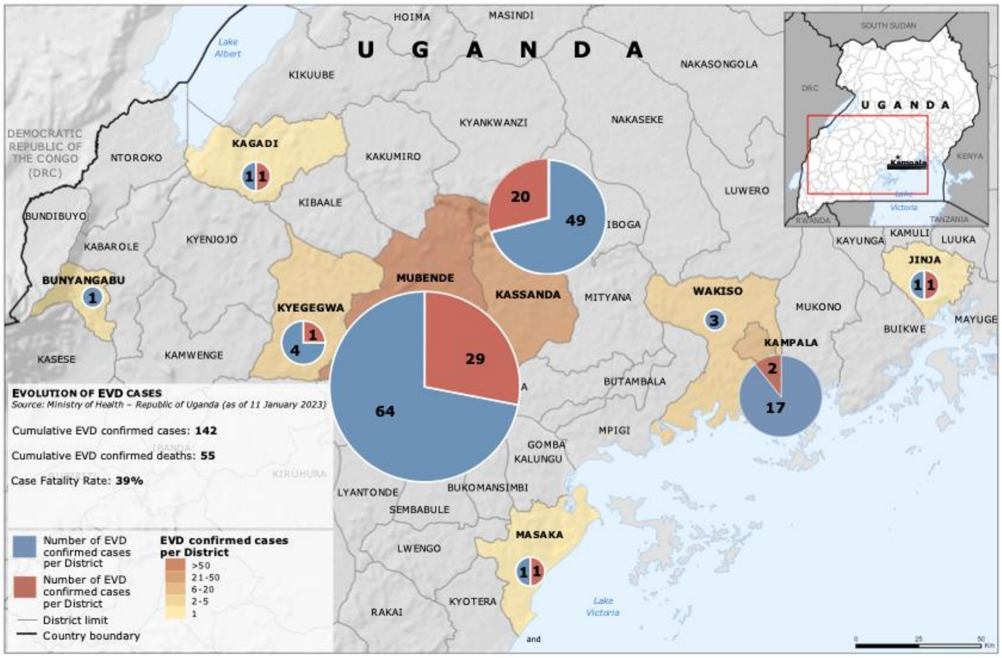


**Supplementary Figure 4. Geographical distribution of EVD cases in Uganda, 2022. Source: Figure adapted from Fig. "Uganda | Ebola Virus Disease (EVD) outbreak update" in [3].**

| District | Confirmed cases | Confirmed deaths | CFR |
|---|---|---|---|
| Bunyangabu | 1 | 0 | NA |
| Jinja | 1 | 1 | 100% |
| Kagadi | 1 | 1 | 100% |
| Kampala | 17 | 2 | 12% |
| Kassanda | 49 | 20 | 41% |
| Kyegegwa | 4 | 1 | 25% |
| Masaka | 1 | 1 | 100% |
| Mubende | 64 | 29 | 45% |
| Wakiso | 4 | 0 | NA |
| Total | 142 | 55 | 39% |

**Supplementary Table 1: Number of cases and deaths (confirmed) of Ebola disease by district, as of 11 January 2023.**

## 3 References


1. CDC. History of Ebola Virus Disease (EVD) Outbreaks. Available online at: https://www.cdc.gov/vhf/ebola/history/chronology.html (accessed on 11 January 2023).

2. World Health Organization Uganda. Ebola virus disease reports. Available online at: https://www.afro.who.int/countries/publications?country=879 (accessed on 11 January 2023).

3. ReliefWeb. Ebola Virus Disease (EVD) outbreak updPdate - DG ECHO Daily Map. ERCC - Emergency Response Coordination Centre. Available online at: https://tinyurl.com/uganda-ebola-map (accessed on 28 November 2022).
